# An empirical comparison of statistical methods for multiple cut-off diagnostic test accuracy meta-analysis of the Edinburgh postnatal depression scale (EPDS) depression screening tool using published results vs individual participant data

**DOI:** 10.1186/s12874-023-02134-w

**Published:** 2024-02-01

**Authors:** Zelalem F. Negeri, Brooke Levis, John P. A. Ioannidis, Brett D. Thombs, Andrea Benedetti, the DEPRESsion Screening Data (DEPRESSD) EPDS Group, Ying Sun, Chen He, Ankur Krishnan, Yin Wu, Parash Mani Bhandari, Dipika Neupane, Mahrukh Imran, Danielle B. Rice, Marleine Azar, Matthew J. Chiovitti, Kira E. Riehm, Jill T. Boruff, Pim Cuijpers, Simon Gilbody, Lorie A. Kloda, Scott B. Patten, Roy C. Ziegelstein, Sarah Markham, Liane Comeau, Nicholas D. Mitchell, Simone N. Vigod, Muideen O. Bakare, Cheryl Tatano Beck, Adomas Bunevicius, Tiago Castro e Couto, Genesis Chorwe-Sungani, Nicolas Favez, Sally Field, Lluïsa Garcia-Esteve, Simone Honikman, Dina Sami Khalifa, Jane Kohlhoff, Laima Kusminskas, Zoltán Kozinszky, Sandra Nakić Radoš, Susan J. Pawlby, Tamsen J. Rochat, Deborah J. Sharp, Johanne Smith-Nielsen, Kuan-Pin Su, Meri Tadinac, S. Darius Tandon, Pavaani Thiagayson, Annamária Töreki, Anna Torres-Giménez, Thandi van Heyningen, Johann M. Vega-Dienstmaier

**Affiliations:** 1 Department of Statistics and Actuarial Science, University of Waterloo, Waterloo, Ontario Canada; 2 Lady Davis Institute for Medical Research, Jewish General Hospital, Montréal, Québec Canada; 3 Department of Epidemiology, Biostatistics, and Occupational Health, McGill University, Montréal, Québec Canada; 4 Department of Medicine, Department of Epidemiology and Population Health, Department of Biomedical Data Science, Department of Statistics, Stanford University, Stanford, CA USA; 5 Department of Psychiatry, McGill University, Montréal, Québec Canada; 6 Department of Medicine, McGill University, Montréal, Québec Canada; 7 Department of Psychology, McGill University, Montréal, Québec Canada; 8 Biomedical Ethics Unit, McGill University, Montréal, Québec Canada; 9 Centre for Outcomes Research & Evaluation, Research Institute of the McGill University Health Centre, Montréal, Québec Canada; 10 Respiratory Epidemiology and Clinical Research Unit, McGill University Health Centre, Montréal, Québec Canada

**Keywords:** Multiple cut-offs meta-analysis, Individual participant data, Depression screening accuracy, Sensitivity, Specificity, Selective reporting bias

## Abstract

**Background:**

Selective reporting of results from only well-performing cut-offs leads to biased estimates of accuracy in primary studies of questionnaire-based screening tools and in meta-analyses that synthesize results. Individual participant data meta-analysis (IPDMA) of sensitivity and specificity at each cut-off via bivariate random-effects models (BREMs) can overcome this problem. However, IPDMA is laborious and depends on the ability to successfully obtain primary datasets, and BREMs ignore the correlation between cut-offs within primary studies.

**Methods:**

We compared the performance of three recent multiple cut-off models developed by Steinhauser et al., Jones et al., and Hoyer and Kuss, that account for missing cut-offs when meta-analyzing diagnostic accuracy studies with multiple cut-offs, to BREMs fitted at each cut-off. We used data from 22 studies of the accuracy of the Edinburgh Postnatal Depression Scale (EPDS; 4475 participants, 758 major depression cases). We fitted each of the three multiple cut-off models and BREMs to a dataset with results from only published cut-offs from each study (published data) and an IPD dataset with results for all cut-offs (full IPD data). We estimated pooled sensitivity and specificity with 95% confidence intervals (CIs) for each cut-off and the area under the curve.

**Results:**

Compared to the BREMs fitted to the full IPD data, the Steinhauser et al., Jones et al., and Hoyer and Kuss models fitted to the published data produced similar receiver operating characteristic curves; though, the Hoyer and Kuss model had lower area under the curve, mainly due to estimating slightly lower sensitivity at lower cut-offs. When fitting the three multiple cut-off models to the full IPD data, a similar pattern of results was observed. Importantly, all models had similar 95% CIs for sensitivity and specificity, and the CI width increased with cut-off levels for sensitivity and decreased with an increasing cut-off for specificity, even the BREMs which treat each cut-off separately.

**Conclusions:**

Multiple cut-off models appear to be the favorable methods when only published data are available. While collecting IPD is expensive and time consuming, IPD can facilitate subgroup analyses that cannot be conducted with published data only.

**Supplementary Information:**

The online version contains supplementary material available at 10.1186/s12874-023-02134-w.

## Background

The accuracy of a screening test when compared with a reference standard is measured by its sensitivity and specificity [1]. For continuous or ordinal tests, sensitivity and specificity are inversely related as a function of the positivity threshold, or cut-off; for tests where higher scores are associated with increased likelihood the underlying target condition is present, as the cut-off is increased, sensitivity decreases, and specificity increases.

For questionnaire-based screening tests, which have ordinal scores and multiple possible cut-offs, authors of primary studies often only report sensitivity and specificity for a standard cut-off or for an “optimal” cut-off that maximizes combined sensitivity and specificity according to a statistical criterion (e.g., Youden’s J) [2]. Sometimes results from other cut-offs close to the standard or optimal cut-off are also reported. This selective cut-off reporting has been shown to positively bias estimates of accuracy of screening tests in primary studies and in meta-analyses that synthesize results from primary studies [2, 3].

Researchers have used several approaches to meta-analyze results from test accuracy studies with missing results for some cut-offs. Some have meta-analyzed studies at one or several cut-offs selected in advance [4] by including reported accuracy estimates at those cut-offs from individual studies [5, 6]; this approach may lead to overestimated accuracy, however, if primary studies selected the cut-offs to report based on maximized test accuracy [2]. Others have combined primary studies using accuracy estimates from a single cut-off from each primary study, presumably the best-performing cut-off, combining results from different cut-offs across studies [7]; this method would also lead to even greater bias and to a clinically meaningless summary receiver operating characteristic (SROC) curve and combined accuracy estimates [8]. More recently, individual participant data meta-analyses (IPDMA) [9–12], have evaluated sensitivity and specificity at each cut-off, separately, using the bivariate random-effects model (BREM) of Chu and Cole [13], as discussed in Riley et al. [14, 15], which overcomes the selective cut-off bias problem but ignores correlations between cut-offs within the same primary study.

Statistical methods [16–19] that take the correlation between cut-offs into consideration and do not require the same number of cut-offs or identical cut-off values to be reported in each primary study have recently been proposed to simultaneously model data from multiple cut-offs in diagnostic test accuracy studies. Steinhauser et al. [16] proposed a class of linear mixed-effects models to model negative or positive test results as a linear function of cut-offs. Hoyer et al. [17] proposed approaches based on survival methods that are random-effects models and consider missing cut-offs between two observed cut-offs as interval censored. Jones et al. [18] proposed, in a Bayesian framework, a generalised nonlinear mixed model based on multinomial likelihood that employs a Box-Cox or logarithmic transformation to describe the underlying distribution of a continuous biomarker. Most recently, Hoyer and Kuss [19] extended Hoyer et al.’s method [17] by suggesting the family of generalized *F* distributions for describing the distribution of screening test scores.

Recently, Benedetti et al. [20] compared the performance of BREMs [13], Steinhauser et al. [16], and Jones et al. [18] methods when applied to data consisting of published primary study results with missing data for some cut-offs versus individual participant data (IPD) with complete cut-off data for a commonly used depression screening tool, the Patient Health Questionnaire-9 (PHQ-9; 45 studies, 15,020 participants, 1972 major depression cases). The PHQ-9 uses a standard cut-off of ≥10 to detect major depression, and missing cut-offs in primary studies tended to be scattered symmetrically around this standard cut-off. When applied to published data with missing cut-offs, the Steinhauser et al. [16] and Jones et al. [18] models performed better than the BREMs [13] in terms of their ability to recover the full receiver operating characteristics (ROC) curve – which unlike the SROC curve uses the separate cut-offs instead of the primary studies in the meta-analysis as a unit of analysis – from the full IPD. When all methods were applied to the full IPD, the Steinhauser et al. [16] and Jones et al. [18] methods produced similar areas under the curve (AUC) and ROC curves as the BREMs [13], but pooled sensitivity and specificity estimates were slightly lower than those from the BREMs [13].

The aim of the present study was to empirically compare three multiple cut-off models – the Steinhauser et al. [16], Jones et al. [18], and recently proposed Hoyer and Kuss [19] (which was not included in Benedetti et al. [20]) models – to conducting BREMs [13] at each cut-off separately using data from primary studies that assessed the screening accuracy of the Edinburgh Postnatal Depression Scale (EPDS). Unlike the PHQ-9, the EPDS does not have a single standard cut-off, and cut-offs from ≥10 to ≥13 are sometimes used; therefore, the distribution of missing cut-offs may be less symmetrical around a single cut-off [3]. Unlike the study of Zapf et al. [21], that considered the Hoyer et al. [17] model, we aimed to [1] use the latest, generalized, and better-performing model of Hoyer and Kuss [19], and [2] compare the multiple cut-off methods applied to published individual study results with missing cut-offs data to the BREM applied to IPD with complete cut-off data, in the context of diagnostic accuracy studies of depression screening tools. First, to replicate standard meta-analytic practice and compare it to IPDMA, we fitted BREMs to published cut-off results and compared results with BREMs fitted to the full IPD dataset for all relevant cut-offs. Second, to compare the ability of the multiple cut-off methods to recover the ROC curve from the full IPD dataset, we compared the multiple cut-off models when applied to published primary study results with missing data for some cut-offs to BREMs applied to the full IPD with results for all cut-offs. Third, we compared the three multiple cut-off models and BREMs when applied to the full IPD to describe each model’s performance in the absence of missing cut-offs. Fourth, we fitted the three multiple cut-off models to both the full IPD dataset and to published primary study results and compared results across models to evaluate differences between the models due to data types.

## Methods

This study uses data from an IPDMA of the accuracy of the EPDS for screening to detect major depression among pregnant and postpartum women [12]. A PROSPERO registration (CRD42015024785) and a published protocol [22] were available for the original IPDMA. The present study was not included in the original IPDMA protocol, but a separate protocol was prepared and posted on the Open Science Framework (https://osf.io/5hf6t/) prior to study initiation. Because of the overlap of methods in the present study with methods from previous studies, we adopted those methods, including the description of our data and data collection methods [3, 12] and descriptions of the statistical models we compared, which were described in Benedetti et al. [20] (except the Hoyer and Kuss model [19]). We followed guidance from the Text Recycling Research Project [23].

### Identification of eligible studies for the main IPDMA

Eligibility criteria for the main IPDMA of the EPDS were based on how screening would occur in practice. In this article, the same eligibility standards as the main IPDMA of the EPDS were used [12], including administration of the EPDS and a validated diagnostic interview – that identified diagnostic classifications for current Major Depressive Disorder (MDD) or Major Depressive Episode (MDE) – within 2 weeks of each other. If the original data allowed for the identification of eligible participants, datasets where not all participants were eligible were included [12]. Our criteria for defining major depression also followed that of Levis et al. [12] and Benedetti et al. [20].

### Search strategy and study selection

A medical librarian, using a peer-reviewed search strategy [24], searched Medline, Medline In-Process & Other Non-Indexed Citations and PsycINFO via OvidSP, and Web of Science via ISI Web of Knowledge from inception to October 3, 2018. The complete search strategy was published with the original IPDMA [12]. We also reviewed reference lists of relevant reviews and queried contributing authors about non-published studies. Search results were uploaded into RefWorks (RefWorks-COS, Bethesda, MD, USA). After de-duplication, unique citations were uploaded into DistillerSR (Evidence Partners, Ottawa, Canada) for storing and tracking search results.

Two investigators independently reviewed titles and abstracts for eligibility. If either reviewer deemed a study potentially eligible, full-text article review was done by two investigators, independently, with disagreements resolved by consensus, including a third investigator, as necessary.

### Data contribution and synthesis

De-identified original data contributions from authors of suitable datasets were requested [12]. Data at the participant level included EPDS score and the presence or absence of major depression. We applied the supplied weights when datasets had necessary statistical weighting to account for sampling techniques, and we created the necessary weights based on inverse selection probabilities in cases where the original study did not weight [12].

### Data used in the present study

Since the purpose of the present study was to compare statistical methods for multiple cut-off meta-analysis using published data versus IPD, we required that included studies for the present analysis published sensitivity and specificity for at least one cut-off in addition to meeting the inclusion and exclusion criteria for the main IPDMA. We did not consider any data from published studies for which the IPD could not be retrieved. Consistent with our previous work [3], to make the data close enough to the actual data used in the original reports, we excluded studies for which the difference in sample size or major depression cases between the published data and our IPD exceeded 10%. We also excluded studies if they reported diagnostic accuracy for a broader diagnostic category than major depression (e.g., any mood disorder) if diagnoses other than major depression comprised more than 10% of cases. For the eligible data, we constructed a dataset composed of 2 × 2 tables (true positives, false positives, true negatives, false negatives) for only published cut-offs for each study, and we refer to this as the *published dataset*. We refer to the dataset that included results for all cut-offs for each eligible study, rather than just published cut-offs, as the *full IPD dataset*.

### Differences between primary study results, *full IPD dataset*, and *published dataset*

Because of the criteria for inclusion and exclusion criteria employed in our EPDS IPDMA [12], data that had previously been included in the published main studies occasionally differed from those used in the current analysis. First, rather than applying the eligibility standards for the EPDS IPDMA [12] at the study level, they were consistently applied to all participants. Due to this, a subset of the individuals in some of the original studies matched the inclusion requirements for the EPDS *full IPD dataset*. For instance, we only included data from individuals who completed the EPDS and reference standard within a two-week time frame, for adult women who completed assessments while pregnant or within a year of giving birth, and for individuals who were not recruited because they were undergoing psychiatric evaluation or treatment or suspected of having a depressive disorder. Participants who fulfilled these requirements were included from every primary study, while those who failed to, were not [12]. Second, we defined the outcome as “major depression.” Some original studies, nevertheless, provided accuracy scores for diagnoses of depression wider than major depression, including “major + minor depression” or “any depressive disorder.” Third, we created suitable weights based on inverse selection probabilities for cases where sampling techniques called for weighting, but the primary study did not. This happened, for example, when the diagnostic interview was given to all those who received positive screening results but only to a randomly selected group of individuals with negative screening results [12]. Fourth, we compared findings calculated using the raw datasets with published information on participants and diagnostic accuracy outcomes during our data validation procedure. We detected and fixed errors in conjunction with the primary research investigators where the primary data that we obtained from the investigators and the original publications conflicted [12]. After making the aforementioned changes and exclusions for the *published dataset*, we only estimated specificity and sensitivity for the cut-offs that were included in the original studies [20].

### Statistical analyses

First, to replicate conventional meta-analytic practice, we fitted BREMs [13] to the *published dataset*, separately for each cut-off, and obtained pooled sensitivity and specificity with 95% confidence intervals (CIs). We evaluated results for all possible EPDS cut-offs (0 to 30) and presented results for those in a clinically relevant range (7 to 15) as we did in our main EPDS IPDMA [12]. We compared these results to BREMs using IPDMA with data from the *full IPD dataset*.

Second, we fitted the three multiple cut-off methods (i.e., the Steinhauser et al. [16], Jones et al. [18], and Hoyer and Kuss [19] models) to the *published dataset* and compared to the BREMs [13] fitted to the *full IPD dataset* to evaluate how well each model recovered the ROC curve from the full IPD.

Third, we fitted the three multiple cut-off models [16, 18, 19] to the *full IPD dataset* and compared results across these models and the BREMs [13], also applied to the *full IPD dataset*, to assess whether any differences in results were due to differences in modelling approaches instead of differences in data type (published data with missing cut-offs versus full IPD).

Fourth, to evaluate whether differences in results were due to data types, we compared results across the three multiple cut-off models [16, 18, 19] applied to both the *full IPD dataset* and to the *published dataset*.

The BREM [13] is a two-stage random-effects meta-analytic approach that estimates pooled logit-transformed sensitivity and specificity simultaneously, accounting for the correlation between sensitivity and specificity across studies and for the precision by which sensitivity and specificity are estimated within studies. The BREM is fitted separately at each cut-off. It does not account for the correlation across cut-offs within a study or make any assumptions about the shape of the association between cut-offs and sensitivity or specificity. The AUC of the full ROC curve was obtained by numerical integration based on the trapezoidal rule, and a 95% CI for the AUC was estimated using bootstrap resampled data at the study and participant level [25].

The Steinhauser et al. [16] approach is a bivariate linear mixed-effects approach that models a study-specific logit-transformed proportion of negative test results (1 – sensitivity, specificity) at each cut-off through random-effects to account for the heterogeneity across studies in the meta-analysis. We used restricted maximum likelihood (REML) criteria [26, 27] to choose among the eight linear mixed-effects models proposed by Steinhauser et al. [16], which differ in their random-effects structures. Accordingly, the “different random intercept and different random slope” model [16] was found to fit both the *published dataset* and the *full IPD dataset* well.

The Jones et al. [18] approach is a Bayesian random-effects model that describes the variability in the test results between cut-offs by the exact multinomial distribution. The model assumes the logistic distribution for the distribution of the Box-Cox or natural logarithm transformed test results in cases and non-cases group, and accounts for within-study correlation due to multiple cut-offs. To describe the variation in sensitivity and specificity across studies, the model assumes that the means and scale parameters of the test results in the case and non-case populations follow a quadrivariate normal distribution with a common mean vector of length four and a four-by-four variance-covariance matrix. We fitted the model to both the *full IPD dataset* and the *published dataset* by estimating the Box-Cox transformation parameters directly from the data instead of assuming the log-logistic distribution for the natural logarithm-transformed screening results since the 95% credible intervals for the Box-Cox transformation parameters did not include 0.

Hoyer and Kuss [19] use an accelerated failure time model by assuming positive test results (sensitivity, 1 – specificity) as the events of interest and the screening test scores as an interval-censored time variable. The family of generalized *F* distributions, which includes the Weibull, lognormal, log-logistic, generalized gamma, Burr III, Burr XII, and generalized log-logistic distribution, is used to describe the distribution of the logarithm of screening test scores. In the accelerated failure time framework, after log-transformation of the screening test scores, bivariate normally distributed random intercepts in the linear predictor are used to account for within-study correlation across screening test scores for different cut-offs and to account for the inherent correlation between sensitivity and specificity across studies. Sensitivity and specificity of a test are predicted from the survival functions of the respective distributions at a specified cut-off threshold. The Bayesian Information Criterion (BIC) [28] is used to choose the best-fitting model. Accordingly, the Burr III and the GF models were best fitting and used for the *published dataset* and the *full IPD dataset*, respectively.

For each method and at each step, we estimated cut-off-specific pooled sensitivity and specificity and corresponding 95% CIs and the AUC across the full range of EPDS cut-offs (0 to 30). We compared point estimates, 95% CI widths, and AUC between methods and datasets.

We fitted the BREMs [13], Steinhauser et al. [16], and Jones et al. [18] models in the R [29] programming language via RStudio [30] using the R packages lme4 [27], diagmeta [31], and R2WinBUGS [32], respectively. The Hoyer and Kuss [19] model was fitted in SAS using the NLMIXED procedure to obtain the maximum likelihood estimates of model parameters via the Gauss Hermite quadrature.

## Results

### Search results and dataset inclusion

**Fig. 1:**
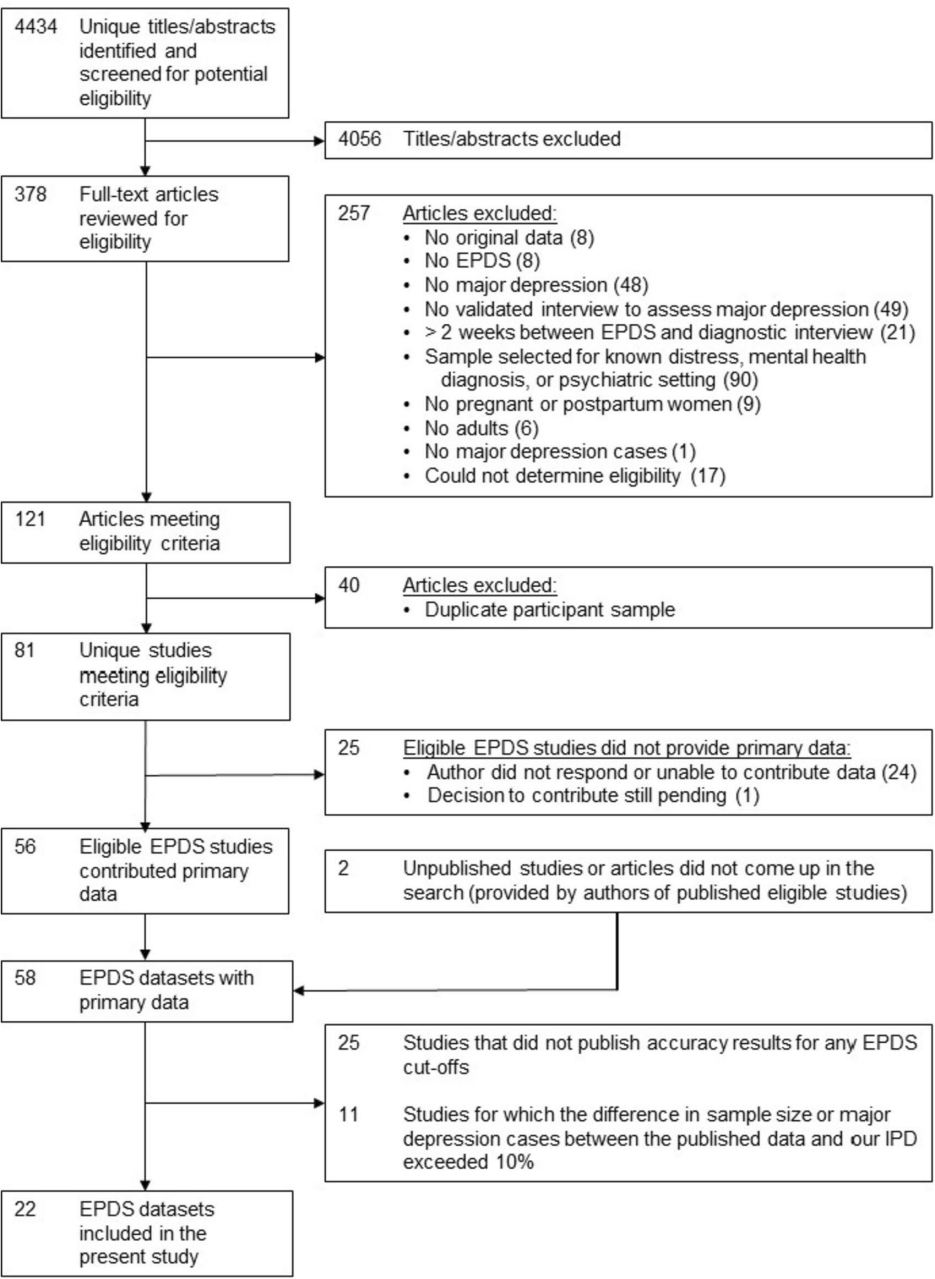
Flow diagram of study selection process

A total of 4434 unique titles and abstracts were identified from database searches; of these, 4056 were excluded after reviewing titles and abstracts and 257 after reviewing full texts, resulting in 121 eligible articles with data from 81 unique participant samples, of which 56 (69%) contributed datasets. Two additional studies were contributed by primary study authors, resulting in a total of 58 studies that provided participant data. We excluded 25 studies that did not publish accuracy results for any EPDS cut-off and 11 studies for which the difference in sample size or number of major depression cases between the published data and our IPD exceeded 10%, leaving data from a total of 22 primary studies that were included in the present study (38% of 58 identified studies that published accuracy results; see Fig. 1).

### Description of included studies

The 22 studies included 4475 participants and 758 major depression cases in the *full IPD dataset*. These numbers vary by cut-off in the *published dataset*, which is a subset of the *full IPD dataset* with results only from cut-offs in the primary studies for which results were published (see Table 1). The aggregate distribution of published EPDS cut-offs by the primary studies included in the *published dataset* is depicted in Appendix Fig. A1. The overall distribution of EPDS scores among participants with and without major depression is shown in Appendix Table A1 and Fig. A2.

**Table 1:** Number of studies, participants, and major depression cases in the *full IPD dataset* and in the *published dataset*

Full IPD dataset				Published dataset		
Cut-off	N Studies	N Participants	N Major Depression Cases	N Studies	N Participants	N Major Depression Cases
7	22	4475	758	9	1829	265
8	22	4475	758	11	2336	337
9	22	4475	758	14	3127	460
10	22	4475	758	13	2631	353
11	22	4475	758	14	2782	395
12	22	4475	758	13	2693	370
13	22	4475	758	18	3398	568
14	22	4475	758	10	2326	265
15	22	4475	758	6	1286	131

### Comparison of sensitivity and specificity

In Appendix Tables A2 to A5 we present the sensitivity and specificity estimates with their corresponding 95% CIs (Steinhauser et al. [16], Hoyer and Kuss [19], BREMs [13]) or credible intervals (Jones et al. [18] model) for both the *published dataset* and *full IPD dataset* for cut-offs 7 to 15.

**Fig. 2:**
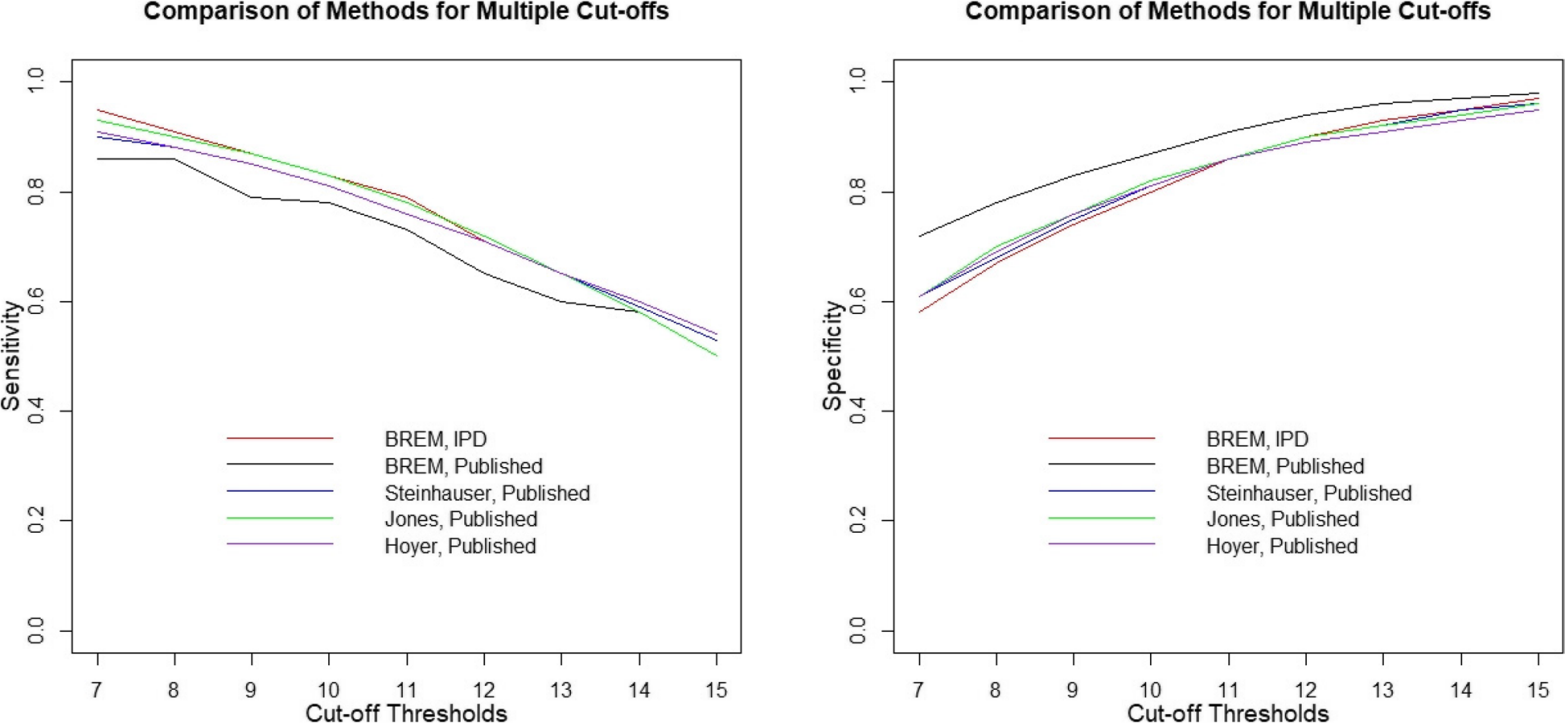
Comparing the sensitivity (left) and specificity (right) estimates when the BREMs [13], Steinhauser et al. [16], Jones et al. [18], and Hoyer and Kuss [19] models were fitted to the published data with when the BREMs [13] were fitted to the full IPD dataset

Figure 2 depicts pooled sensitivity and specificity by cut-off when the BREMs [13], Steinhauser et al. [16], Jones et al. [18], and Hoyer and Kuss [19] models were fitted to the *published dataset* and when the BREMs [13] were fitted to the *full IPD dataset*. The BREMs [13] fitted to the *published dataset* yielded lower sensitivity estimates for most cut-offs compared to the BREMs [13] fitted to the *full IPD dataset*, with mean absolute difference between the two models of 0.05 (range: 0.00 to 0.09). The right-hand panel of Fig. 2 shows that the specificity estimated by the BREMs [13] fitted to the *published dataset* was higher than that estimated by the BREMs [13] fitted to the *full IPD dataset*, and that the difference decreased as the cut-off increased (mean absolute difference: 0.06, range: 0.01 to 0.14).

Compared to the BREMs [13] fitted to the *full IPD dataset*, the Steinhauser et al. [16] and Hoyer and Kuss [19] approaches applied to the *published dataset* had lower sensitivity estimates at lower cut-offs and the same or slightly higher estimates at higher cut-offs, with mean absolute difference of 0.02 (range: 0.00 to 0.05) and 0.02 (range: 0.00 to 0.04), respectively. On the other hand, the Jones et al. [18] model applied to the *published dataset* generated similar sensitivity estimates to the BREMs applied to the full IPD dataset across cut-offs (mean absolute difference: 0.01, range: 0.00 to 0.02). The Steinhauser et al. [16], Hoyer and Kuss [19], and Jones et al. [18] models fitted to the *published dataset* had higher specificity estimates at lower cut-offs but similar or lower estimates for higher cut-offs compared to those estimated by the BREMs [13] fitted to the *full IPD dataset*, with respective mean absolute differences of 0.01 (range: 0.00 to 0.03), 0.02 (range: 0.00 to 0.03), and 0.01 (range: 0.00 to 0.03).

**Fig. 3:**
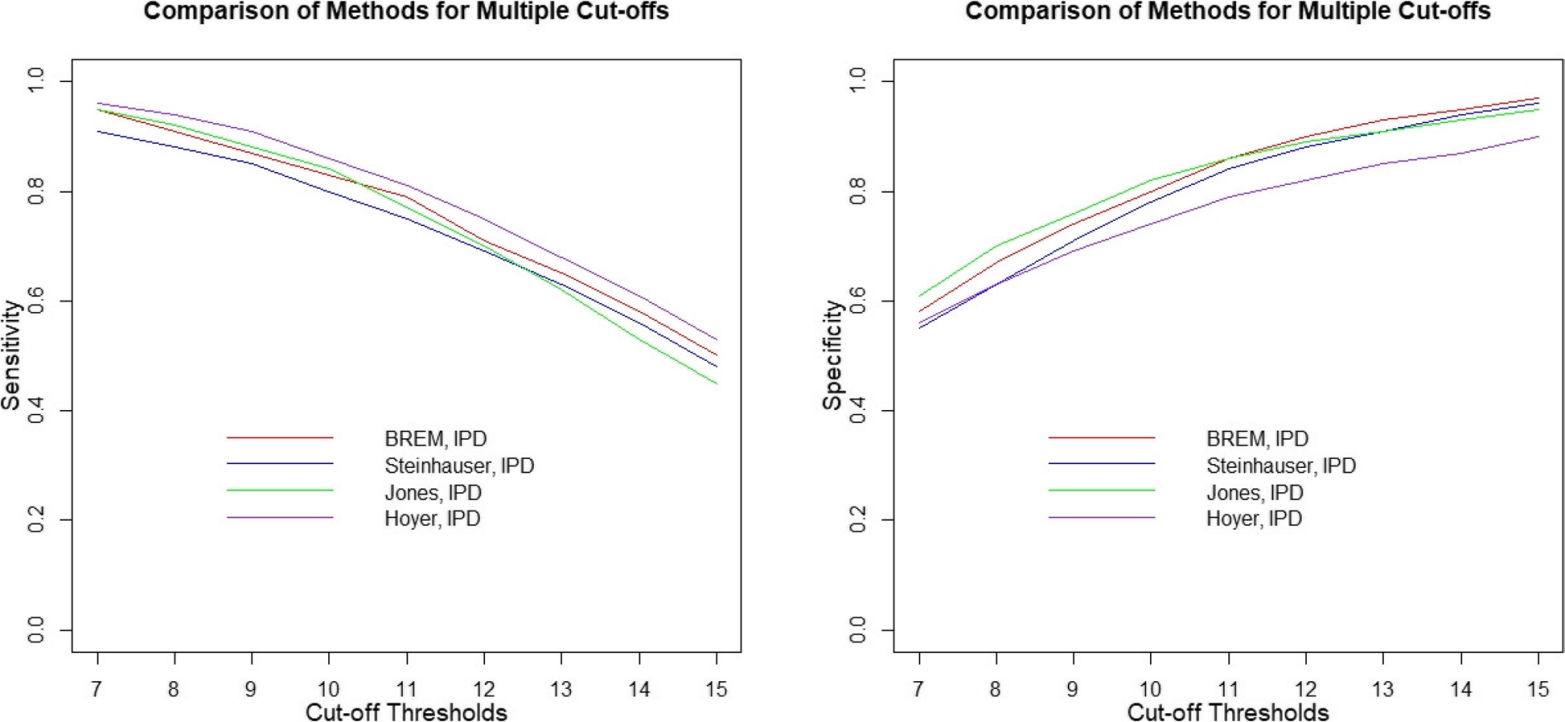
Comparing the sensitivity (left) and specificity (right) estimates when the BREMs [13], Steinhauser et al. [16], Jones et al. [18], and Hoyer and Kuss [19] models were fitted to the full IPD data with when the BREMs [13] were fitted to the full IPD dataset

Figure 3 compares the Steinhauser et al. [16], Jones et al. [18], and Hoyer and Kuss [19] models when fitted to *the full IPD dataset* with the BREMs [13] fitted to the *full IPD dataset*. The Steinhauser et al. [16] model had lower sensitivity (mean absolute difference: 0.03, range: 0.02 to 0.04) and specificity (mean absolute difference: 0.02, range: 0.01 to 0.04) estimates for all cut-offs compared to the BREMs [13]. The sensitivity and specificity estimated by the Jones et al. [18] model were higher or similar at lower cut-offs and lower at higher cut-offs, with a mean absolute difference of 0.02 for sensitivity (range: 0.00 to 0.05) and 0.02 for specificity (range: 0.00 to 0.03). The Hoyer and Kuss [19] model generated estimates of sensitivity that were higher for all cut-offs (mean absolute difference: 0.03, range: 0.01 to 0.04) and estimates of specificity that were lower for all cut-offs (mean absolute difference: 0.06, range: 0.02 to 0.08) compared to estimates generated by the BREMs [13].

Compared to the Steinhauser et al. [16] model fitted to the *full IPD dataset*, the Steinhauser et al. [16] approach applied to the *published dataset* had similar sensitivity estimates at lower cut-offs but higher estimates at upper cut-offs (mean absolute difference: 0.02, range: 0.00 to 0.05), and higher specificity estimates for all cut-offs (mean absolute difference: 0.03, range: 0.00 to 0.06). Compared to the Jones et al. [18] model fitted to the *full IPD dataset*, the Jones et al. [18] model applied to the *published dataset* had lower sensitivity estimates at lower cut-offs and higher estimates at upper cut-offs (mean absolute difference: 0.02, range: 0.01 to 0.05), but similar specificity estimates (mean absolute difference: 0.00, range: 0.00 to 0.01). Compared to the Hoyer and Kuss [19] model fitted to the full IPD, the Hoyer and Kuss [19] model applied to the *published dataset* generated estimates of sensitivity that were lower for all cut-offs except ≥15 (mean absolute difference: 0.04, range: 0.01 to 0.06) and higher estimates of specificity for all cut-offs (mean absolute difference: 0.06, range: 0.05 to 0.07). See Appendix Tables A3 to A5.

### Comparison of confidence or credible interval width

As expected, the widths of the estimated 95% CIs for sensitivity and specificity using the *full IPD dataset* were narrower than those estimated using the *published dataset* for the BREMs [13], (mean absolute difference: 0.07, range: 0.01 to 0.12 for sensitivity; mean absolute difference: 0.02, range: 0.00 to 0.09 for specificity). All four modelling approaches had similar 95% CIs for sensitivity and specificity when applied to the *full IPD dataset*, with an increasing 95% CI width for sensitivity and decreasing 95% CI width for specificity as the cut-offs increased or the number of major depression cases decreased. Although estimated 95% CIs for sensitivity using the *full IPD dataset* were narrower than those estimated using the *published dataset* for the Steinhauser et al. [16] and Hoyer and Kuss [19] models (mean absolute difference: 0.05, range: 0.03 to 0.07 and mean absolute difference: 0.06, range: 0.04 to 0.09, respectively), both models produced similar estimated 95% CIs for specificity when the *published dataset* or the *full IPD dataset* was used, with a mean 95% CI width of ≤0.01 (range: 0.00 to 0.02 for Steinhauser et al. [16], range: 0.00 to 0.03 for Hoyer and Kuss [19]) across all cut-offs. The Jones et al. [18] model, however, yielded similar estimated credible intervals for sensitivity and specificity between the datasets, with a mean absolute difference across cut-offs of 0.002 (range: 0.00 to 0.02) and 0.01 (range: 0.00 to 0.02) for sensitivity and specificity, respectively. (See Appendix Figs. A3 and A4).

### Comparison in terms of ROC curves and AUC

**Fig. 4:**
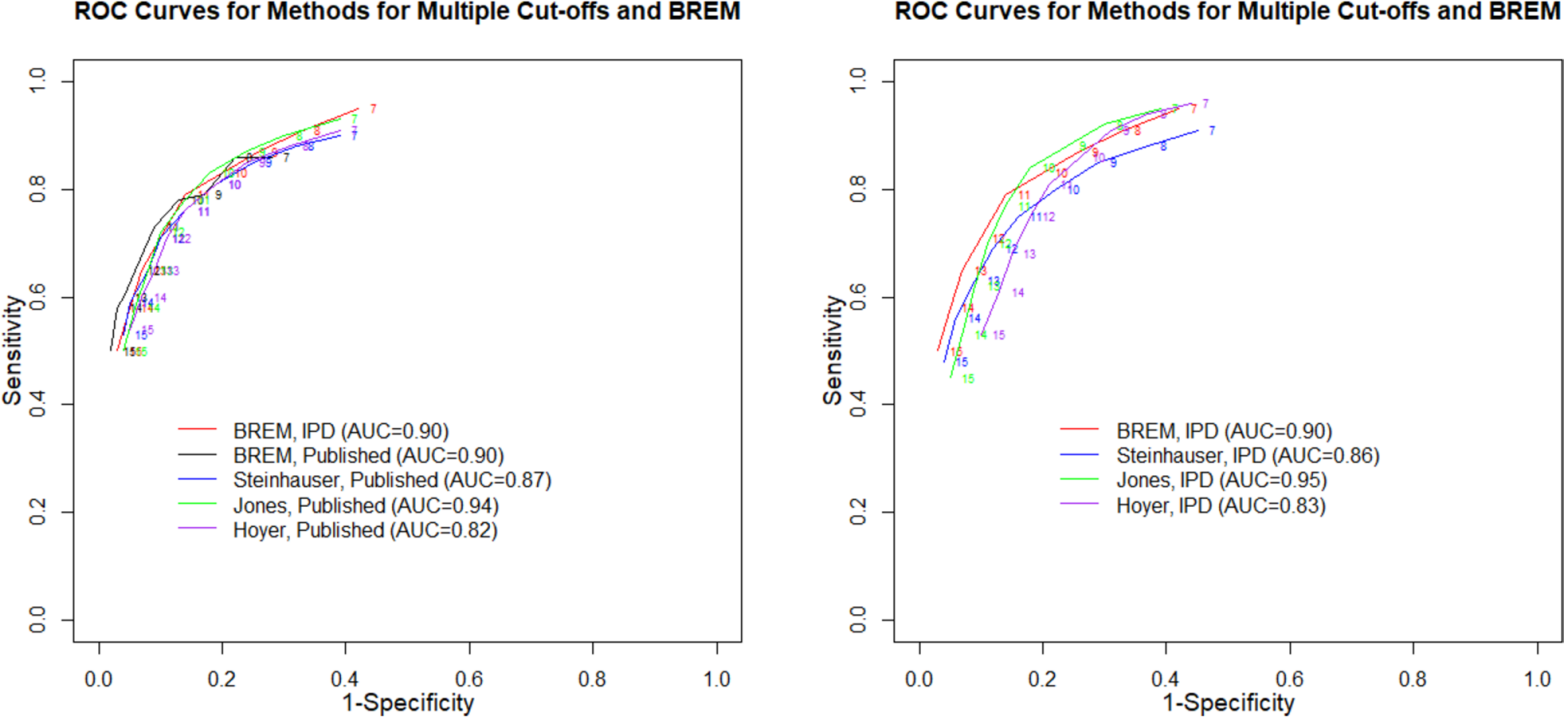
Comparing ROC curves when the BREMs [13], Steinhauser et al. [16], Jones et al. [18], and Hoyer and Kuss [19] approaches were fitted to the published data (left) and full IPD (right) with when the BREMs [13] were fitted to the full IPD dataset

Figure 4 depicts the comparison of the ROC curves of the four modelling approaches when applied to the *published dataset* versus the BREMs [13] applied to the *full IPD dataset* (left panel) and when all approaches were applied to the *full IPD dataset* (right panel).

The AUC of the BREMs [13], Steinhauser et al. [16], Jones et al. [18], and Hoyer and Kuss [19] methods when fitted to the *published dataset* were 0.90, 0.87, 0.94, and 0.82, respectively. The ROC curve from the BREMs [13] fitted to the *published dataset* largely deviated from that fitted to the *full IPD dataset*, whereas the ROC curves from the Steinhauser et al. [16] and Jones et al. [18] approaches fitted to the *published dataset* were similar to the BREMs [13] fitted to the *full IPD dataset*. The Hoyer and Kuss [19] approach resulted in a lower AUC (Fig. 4, left panel).

A similar pattern of results was observed when the approaches were fitted to the *full IPD dataset*, though ROC curves were more spread out. The AUC of the BREMs [13], Steinhauser et al. [16], Jones et al. [18], and Hoyer and Kuss [19] approaches when fitted to the *full IPD dataset* were 0.90, 0.86, 0.95, and 0.83, respectively. Compared to the ROC curve for the BREMs [13], the ROC curves for the Jones et al. [18] and Hoyer and Kuss [19] approaches were lower at lower cut-offs and slightly higher at higher cut-offs. The ROC curve for the Steinhauser et al. [16] approach remained lower than that for the BREMs [13] regardless of the cut-off thresholds (Fig. 4, right panel).

## Discussion

We compared the performance of three recently developed multiple cut-off methods by Steinhauser et al. [16], Jones et al. [18], and Hoyer and Kuss [19] that account for missing cut-offs when meta-analyzing diagnostic test accuracy studies with multiple cut-offs. These methods do not require IPD, which is costly and labour-intensive to collect [33]. We compared them with BREMs [13] when each of the three multiple cut-off models was fitted to both a *published dataset* with missing cut-offs and using IPD from 22 studies on the diagnostic accuracy of the EPDS (the *full IPD dataset*).

Most of the results we found were consistent with the findings of Benedetti et al. [20] The BREMs [13] fitted to the *published dataset* resulted in lower sensitivity and higher specificity estimates for most cut-offs, and a divergent ROC curve with similar AUC compared to results from the BREMs [13] fitted to the *full IPD dataset* (Fig. 2 and Table A2), suggesting that results from the BREMs [13] fitted to published data are biased due to selective cut-off reporting [2, 3].

Compared to the BREMs [13] fitted to the *full IPD dataset*, the Steinhauser et al. [16], Jones et al. [18], and Hoyer and Kuss [19] models fitted to the *published dataset* produced similar ROC curves; though, the Hoyer and Kuss [19] model had lower AUC, mainly due to estimating slightly lower sensitivity at lower cut-offs (Fig. 2). When fitting the three multiple cut-off models to the *full IPD dataset*, a similar pattern of results was observed (Fig. 3). Importantly, all models had similar 95% CIs for sensitivity and specificity, and the CI width increased with cut-off levels for sensitivity and decreased with an increasing cut-off for specificity, even the BREMs which treats each cut-off separately (Tables A2 to A5; Figs. A3 and A4).

The ROC curves estimated by the Hoyer and Kuss model [19] had considerably lower AUC than the Steinhauser et al. [16] and Jones et al. [18] methods (Fig. 4). While this may be due to the sensitivity of the model to starting values, we used an objective statistical approach to choose a starting value that yielded in the best model with the smallest BIC. Moreover, in the simulations presented in Hoyer and Kuss [19], when the Generalized *F* was the true model, the model as specified here underestimated sensitivity and overestimated specificity across cut-offs, similar to the pattern of results seen when this approach was applied to the *full IPD dataset*. For the *published dataset*, this approach estimated the lowest sensitivity at lower cut-offs and highest specificity at upper cut-offs.

The differences in results between the models when fitted to the *full IPD dataset* were likely due to the various assumptions each model makes. Each of the models discussed in this paper assume different distributions to describe the variation in the screening test results. While the recent methods account for the correlation across cut-offs between sensitivities and specificities, the BREM does not. Except the Jones et al. [18] model, which assumes four random-effects, the other models assume only two random-effects to describe the variation in sensitivities and specificities across studies and cut-offs. For example, as pointed out by Benedetti et al. [20], whereas, the Steinhauser et al. [16] model may fit the ROC curve at upper cut-offs where more major depression cases are observed as it assumes a parametric relationship between cut-offs and logit-transformed sensitivities, the Jones et al. [18] model, which additionally assumes the Box-Cox transformation estimated from the data, may recover the true ROC curve better.

The present study showed that recent methods for multiple cut-offs meta-analysis with missing cut-off information are important approaches that can produce reliable estimates in the absence of IPD, unlike standard BREMs [13] at each cut-off separately, which may produce misleading results when there is substantial missingness in reported results at different cut-offs.

We did not find substantial differences between our findings and those of Benedetti et al. [20], suggesting that the recent multiple cut-off models are robust to variations in data characteristics, although further research, including studies with simulated datasets, is needed. Whereas we fitted the models to the EPDS data that consisted of IPD from 22 studies, 4475 participants and 758 major depression cases (Table 1), Benedetti et al. [20] applied the models to the PHQ-9 data that comprised IPD from 45 studies, 15,020 participants and 972 major depression cases. There is also appreciable difference in the distribution of the published data for the cut-offs 7 to 15 (Table A1; Fig. A2), which were used in both studies. Whereas the distribution of missing cut-offs was scattered symmetrically around the standard cut-off of ≥10 for the PHQ-9, the distribution was less symmetrical around the commonly used cut-off of ≥13 for the EPDS (Fig. A1).

Strengths of the present study include assessing the most recent approach of Hoyer and Kuss [19] in addition to those evaluated by Benedetti et al. [20] and the ability to compare results from a dataset with missing cut-offs to IPD that consisted of line-by-line participant data. Additionally, our ability to replicate the findings on Benedetti et al. [20] on a different dataset with differing characteristics supports the best-practice standards for developing knowledge through replication of existing studies using multiple empirical replication studies [34]. A main limitation is the lack of a simulation study upon which the methods can be examined using true population parameters instead of empirical data, although the in-progress simulation study as promised by Zapf et al. [21] is anticipated to shed some light on this end. Moreover, we could not include data from 36 (62%) of 58 identified studies that published accuracy results.

## Conclusion

Despite the differences in model assumptions, all three recent methods for multiple cut-off diagnostic data meta-analysis, particularly the Jones et al. [18] model, satisfactorily recovered the ROC curve from the full IPD while being fitted to only the published data with missing cut-offs, which demonstrates the importance of such methods in the absence of IPD. Our results suggest that there is not a substantive disadvantage compared to applying the BREMs to the full IPD. Furthermore, our results suggest that multiple cut-off models are effective methods for meta-analysis of diagnostic test accuracy of depression screening tools when only published data are available, although our results may not hold in datasets with very different characteristics. However, we note that collecting full IPD allows additional analyses not possible when only aggregate data are collected (such as, e.g., conducting subgroup analyses). it is important to note that collecting IPD remains an attractive option. Beyond reducing bias from selective cut-off reporting, it can reduce heterogeneity among included studies as it allows for analysis based on predetermined inclusion/exclusion criteria, and it allows for subgroup analysis by important participant characteristics for which primary studies may not have reported results for, which would not be possible using the multiple cut-off models.

### Supplementary Information

**Additional file 1: Table A1. **Distribution of EPDS scores by cut-off among participants with depression and without depression. **Table A2. **Estimated sensitivity and specificity, 95% confidence intervals (CI) and CI widths for each cut-off when BREM [13] was fitted to the published and full IPD dataset. **Table A3. **Estimated sensitivity and specificity, 95% confidence intervals (CI) and CI widths for each cut-off when Steinhauser et al. [16] model was fitted to the published and full IPD dataset. **Table A4. **Estimated sensitivity and specificity, 95% confidence intervals (CI) and CI widths for each cut-off when Jones et al. [18] model is fit to the published (top) and full IPD (bottom) dataset.** Table A5. **Estimated sensitivity and specificity, 95% confidence intervals (CI) and CI widths for each cut-off when Hoyer and Kuss [19] model is fit to the published (top) and full IPD (bottom) dataset.** Figure A1. **Distribution of published EPDS cut-offs by the number of primary studies included in the meta-analyses using the published dataset. **Figure A2. **Distribution of EPDS scores among participants with depression (red) and without depression (blue). Purple portions are part of both the blue and red distributions.** Figure A3. **Estimated sensitivity (left) and specificity (right) and 95% Confidence Interval (Credible Interval for Jones et al. [18]) by cut-off for the BREM [13], Steinhauser et al. [16], Jones et al. [18] and Hoyer and Kuss [19] methods applied to the full IPD dataset. **Figure A4. **Estimated sensitivity (left) and specificity (right) and 95% Confidence Interval (Credible Interval for Jones et al. [18]) by cut-off for the BREM [13], Steinhauser et al. [16], Jones et al. [18] and Hoyer and Kuss [19] methods applied to the published dataset.

## Data Availability

The data that support the findings of this study are available upon reasonable request from the corresponding author. The data are not publicly available due to privacy or ethical restrictions in agreements with individual data contributors.
