# Supplementary material for: An empirical comparison of statistical methods for multiple cut-off diagnostic test accuracy meta-analysis of the Edinburgh postnatal depression scale (EPDS) depression screening tool using published results vs individual participant data
Source: BMC Med Res Methodol. 2024 Feb 1;24:28. doi: 10.1186/s12874-023-02134-w (PMC10832258; doi:10.1186/s12874-023-02134-w)
Supplement: Supplementary file 1 — Additional file 1: Table A1. Distribution of EPDS scores by cut-off among participants with depression and without depression. Table A2. Estimated sensitivity and specificity, 95% confidence intervals (CI) and CI widths for each cut-off when BREM [13] was fitted to the published and full IPD dataset. Table A3. Estimated sensitivity and specificity, 95% confidence intervals (CI) and CI widths for each cut-off when Steinhauser et al. [16] model was fitted to the published and full IPD dataset. Table A4. Estimated sensitivity and specificity, 95% confidence intervals (CI) and CI widths for each cut-off when Jones et al. [18] model is fit to the published (top) and full IPD (bottom) dataset. Table A5. Estimated sensitivity and specificity, 95% confidence intervals (CI) and CI widths for each cut-off when Hoyer and Kuss [19] model is fit to the published (top) and full IPD (bottom) dataset. Figure A1. Distribution of published EPDS cut-offs by the number of primary studies included in the meta-analyses using the published dataset. Figure A2. Distribution of EPDS scores among participants with depression (red) and without depression (blue). Purple portions are part of both the blue and red distributions. Figure A3. Estimated sensitivity (left) and specificity (right) and 95% Confidence Interval (Credible Interval for Jones et al. [18]) by cut-off for the BREM [13], Steinhauser et al. [16], Jones et al. [18] and Hoyer and Kuss [19] methods applied to the full IPD dataset. Figure A4. Estimated sensitivity (left) and specificity (right) and 95% Confidence Interval (Credible Interval for Jones et al. [18]) by cut-off for the BREM [13], Steinhauser et al. [16], Jones et al. [18] and Hoyer and Kuss [19] methods applied to the published dataset. [file 12874_2023_2134_MOESM1_ESM.docx]

**APPENDIX**

Table A1: Distribution of EPDS scores by cut-off among participants with depression and without depression.

Table A2: Estimated sensitivity and specificity, 95% confidence intervals (CI) and CI widths for each cut-off when BREM^13^ was fitted to the published and full IPD dataset.

Table A3: Estimated sensitivity and specificity, 95% confidence intervals (CI) and CI widths for each cut-off when Steinhauser et al.^16^ model was fitted to the published and full IPD dataset.

Table A4: Estimated sensitivity and specificity, 95% confidence intervals (CI) and CI widths for each cut-off when Jones et al.^18^ model is fit to the published (top) and full IPD (bottom) dataset.

Table A5: Estimated sensitivity and specificity, 95% confidence intervals (CI) and CI widths for each cut-off when Hoyer and Kuss^19^ model is fit to the published (top) and full IPD (bottom) dataset.

Figure A1: Distribution of published EPDS cut-offs by the number of primary studies included in the meta-analyses using the *published dataset*.

Figure A2: Distribution of EPDS scores among participants with depression (red) and without depression (blue). Purple portions are part of both the blue and red distributions.

Figure A3: Estimated sensitivity (left) and specificity (right) and 95% Confidence Interval (Credible Interval for Jones et al.^18^) by cut-off for the BREM,^13^ Steinhauser et al.,^16^ Jones et al.^18^ and Hoyer and Kuss^19^ methods applied to the full IPD dataset.

Figure A4: Estimated sensitivity (left) and specificity (right) and 95% Confidence Interval (Credible Interval for Jones et al.^18^) by cut-off for the BREM,^13^ Steinhauser et al.,^16^ Jones et al.^18^ and Hoyer and Kuss^19^ methods applied to the published dataset.

Table A1: Distribution of EPDS scores by cut-off among participants with depression and without depression.

| **Cut-off** | **N With Depression** | **N Without Depression** |
| --- | --- | --- |
| 0  1  2  3  4  5  6  7 | 9  16  15  13  23  28  49  54 | 1564  1093  1311  1113  1125  999  991  826 |
| 8 | 58 | 772 |
| 9 | 63 | 751 |
| 10 | 66 | 623 |
| 11 | 136 | 496 |
| 12 | 132 | 426 |
| 13 | 138 | 344 |
| 14  15 | 153  168 | 264  223 |
| 16  17 | 148  142 | 170  111 |
| 18 | 136 | 107 |
| 19 | 120 | 62 |
| 20 | 79 | 33 |
| 21 | 79 | 33 |
| 22 | 69 | 15 |
| 23 | 60 | 12 |
| 24 | 39 | 7 |
| 25  26  27  28  29  30 | 27  17  11  12  8  1 | 9  4  3  1  0  0 |

Table A2: Estimated sensitivity and specificity, 95% confidence intervals (CI) and CI widths for each cut-off when BREM^13^ was fitted to the published and full IPD dataset.

| **BREM^13^ fitted to *published data*** | | | | | | | | | |
| --- | --- | --- | --- | --- | --- | --- | --- | --- | --- |
| **Cut-off** | **N**  **Studies** | **N**  **Participants** | **N**  **MDD Cases** | **Sensitivity** | **(95% CI)** | **Sensitivity CI Width** | **Specificity** | **(95% CI)** | **Specificity CI Width** |
| 7 | 9 | 1829 | 265 | 0.86 | (0.78, 0.91) | 0.13 | 0.72 | (0.59, 0.83) | 0.24 |
| 8 | 11 | 2336 | 337 | 0.86 | (0.79, 0.91) | 0.12 | 0.78 | (0.68, 0.85) | 0.17 |
| 9 | 14 | 3127 | 460 | 0.79 | (0.68, 0.87) | 0.19 | 0.83 | (0.77, 0.88) | 0.11 |
| 10 | 13 | 2631 | 353 | 0.78 | (0.64, 0.88) | 0.24 | 0.87 | (0.81, 0.92) | 0.11 |
| 11 | 14 | 2782 | 395 | 0.73 | (0.60, 0.82) | 0.22 | 0.91 | (0.85, 0.95) | 0.10 |
| 12 | 13 | 2693 | 370 | 0.65 | (0.51, 0.76) | 0.25 | 0.94 | (0.89, 0.96) | 0.07 |
| 13 | 18 | 3398 | 568 | 0.60 | (0.51, 0.69) | 0.18 | 0.96 | (0.93, 0.98) | 0.05 |
| 14 | 10 | 2326 | 265 | 0.58 | (0.46, 0.69) | 0.23 | 0.97 | (0.92, 0.99) | 0.07 |
| 15 | 6 | 1286 | 131 | 0.50 | (0.38, 0.62) | 0.24 | 0.98 | (0.94, 1.00) | 0.06 |
| **BREM^13^ fitted to *full IPD*** | | | | | | | | | |
| **Cut-off** | **N**  **Studies** | **N**  **Participants** | **N**  **MDD Cases** | **Sensitivity** | **(95% CI)** | **Sensitivity CI Width** | **Specificity** | **(95% CI)** | **Specificity CI Width** |
| 7 | 22 | 4475 | 758 | 0.95 | (0.90, 0.97) | 0.07 | 0.58 | (0.50, 0.65) | 0.15 |
| 8 | 22 | 4475 | 758 | 0.91 | (0.85, 0.94) | 0.09 | 0.67 | (0.59, 0.73) | 0.14 |
| 9 | 22 | 4475 | 758 | 0.87 | (0.82, 0.91) | 0.09 | 0.74 | (0.67, 0.80) | 0.13 |
| 10 | 22 | 4475 | 758 | 0.83 | (0.76, 0.88) | 0.12 | 0.80 | (0.75, 0.85) | 0.10 |
| 11 | 22 | 4475 | 758 | 0.79 | (0.72, 0.85) | 0.13 | 0.86 | (0.81, 0.90) | 0.09 |
| 12 | 22 | 4475 | 758 | 0.71 | (0.63, 0.78) | 0.15 | 0.90 | (0.86, 0.93) | 0.07 |
| 13 | 22 | 4475 | 758 | 0.65 | (0.56, 0.73) | 0.17 | 0.93 | (0.90, 0.96) | 0.06 |
| 14 | 22 | 4475 | 758 | 0.58 | (0.49, 0.67) | 0.18 | 0.95 | (0.93, 0.97) | 0.04 |
| 15 | 22 | 4475 | 758 | 0.50 | (0.42, 0.58) | 0.16 | 0.97 | (0.95, 0.98) | 0.03 |

Table A3: Estimated sensitivity and specificity, 95% confidence intervals (CI) and CI widths for each cut-off when Steinhauser et al.^16^ model was fitted to the published and full IPD dataset.

| **Steinhauser et al.^16^ fitted to *published data*** | | | | | | | | | |
| --- | --- | --- | --- | --- | --- | --- | --- | --- | --- |
| **Cut-off** | **N**  **Studies** | **N**  **Participants** | **N**  **MDD Cases** | **Sensitivity** | **(95% CI)** | **Sensitivity CI Width** | **Specificity** | **(95% CI)** | **Specificity CI Width** |
| 7 | 9 | 1829 | 265 | 0.90 | (0.83, 0.95) | 0.12 | 0.61 | (0.52, 0.69) | 0.17 |
| 8 | 11 | 2336 | 337 | 0.88 | (0.80, 0.93) | 0.13 | 0.68 | (0.60, 0.75) | 0.15 |
| 9 | 14 | 3127 | 460 | 0.85 | (0.76, 0.91) | 0.15 | 0.75 | (0.68, 0.81) | 0.13 |
| 10 | 13 | 2631 | 353 | 0.81 | (0.71, 0.88) | 0.17 | 0.81 | (0.75, 0.86) | 0.11 |
| 11 | 14 | 2782 | 395 | 0.76 | (0.66, 0.84) | 0.18 | 0.86 | (0.81, 0.90) | 0.09 |
| 12 | 13 | 2693 | 370 | 0.71 | (0.60, 0.80) | 0.20 | 0.90 | (0.85, 0.93) | 0.08 |
| 13 | 18 | 3398 | 568 | 0.65 | (0.54, 0.76) | 0.22 | 0.92 | (0.89, 0.95) | 0.06 |
| 14 | 10 | 2326 | 265 | 0.59 | (0.48, 0.70) | 0.22 | 0.95 | (0.92, 0.96) | 0.04 |
| 15 | 6 | 1286 | 131 | 0.53 | (0.41, 0.64) | 0.23 | 0.96 | (0.94, 0.97) | 0.03 |
| **Steinhauser et al.^16^ fitted to *full IPD*** | | | | | | | | | |
| **Cut-off** | **N**  **Studies** | **N**  **Participants** | **N**  **MDD Cases** | **Sensitivity** | **(95% CI)** | **Sensitivity CI Width** | **Specificity** | **(95% CI)** | **Specificity CI Width** |
| 7 | 22 | 4475 | 758 | 0.91 | (0.86, 0.94) | 0.08 | 0.55 | (0.47, 0.62) | 0.15 |
| 8 | 22 | 4475 | 758 | 0.88 | (0.83, 0.92) | 0.09 | 0.63 | (0.56, 0.70) | 0.14 |
| 9 | 22 | 4475 | 758 | 0.85 | (0.78, 0.89) | 0.11 | 0.71 | (0.64, 0.77) | 0.13 |
| 10 | 22 | 4475 | 758 | 0.80 | (0.73, 0.86) | 0.13 | 0.78 | (0.72, 0.83) | 0.11 |
| 11 | 22 | 4475 | 758 | 0.75 | (0.67, 0.82) | 0.15 | 0.84 | (0.79, 0.88) | 0.09 |
| 12 | 22 | 4475 | 758 | 0.69 | (0.61, 0.77) | 0.16 | 0.88 | (0.84, 0.91) | 0.07 |
| 13 | 22 | 4475 | 758 | 0.63 | (0.54, 0.70) | 0.16 | 0.91 | (0.88, 0.94) | 0.06 |
| 14 | 22 | 4475 | 758 | 0.56 | (0.47, 0.64) | 0.17 | 0.94 | (0.91, 0.96) | 0.05 |
| 15 | 22 | 4475 | 758 | 0.48 | (0.40, 0.56) | 0.16 | 0.96 | (0.94, 0.97) | 0.03 |

Table A4: Estimated sensitivity and specificity, 95% confidence intervals (CI) and CI widths for each cut-off when Jones et al.^18^ model is fit to the published (top) and full IPD (bottom) dataset.

| **Jones et al.^18^ fit to *Published data*** | | | | | | | | | |
| --- | --- | --- | --- | --- | --- | --- | --- | --- | --- |
| **Cut-off** | **N**  **Studies** | **N**  **Participants** | **N**  **MDD Cases** | **Sensitivity** | **(95% CI)** | **Sensitivity CI Width** | **Specificity** | **(95% CI)** | **Specificity CI Width** |
| 7 | 9 | 1829 | 265 | 0.93 | (0.88, 0.96) | 0.08 | 0.61 | (0.52, 0.70) | 0.18 |
| 8 | 11 | 2336 | 337 | 0.90 | (0.84, 0.95) | 0.11 | 0.70 | (0.61, 0.77) | 0.16 |
| 9 | 14 | 3127 | 460 | 0.87 | (0.80, 0.92) | 0.12 | 0.76 | (0.69, 0.83) | 0.14 |
| 10 | 13 | 2631 | 353 | 0.83 | (0.76, 0.89) | 0.13 | 0.82 | (0.76, 0.87) | 0.11 |
| 11 | 14 | 2782 | 395 | 0.78 | (0.70, 0.85) | 0.15 | 0.86 | (0.81, 0.90) | 0.09 |
| 12 | 13 | 2693 | 370 | 0.72 | (0.63, 0.80) | 0.17 | 0.90 | (0.86, 0.93) | 0.07 |
| 13 | 18 | 3398 | 568 | 0.65 | (0.56, 0.74) | 0.18 | 0.92 | (0.89, 0.95) | 0.06 |
| 14 | 10 | 2326 | 265 | 0.58 | (0.48, 0.67) | 0.19 | 0.94 | (0.91, 0.96) | 0.05 |
| 15 | 6 | 1286 | 131 | 0.50 | (0.40, 0.60) | 0.20 | 0.96 | (0.93, 0.97) | 0.04 |
| **Jones et al.^18^ fit to *full IPD*** | | | | | | | | | |
| **Cut-off** | **N**  **Studies** | **N**  **Participants** | **N**  **MDD Cases** | **Sensitivity** | **(95% CI)** | **Sensitivity CI Width** | **Specificity** | **(95% CI)** | **Specificity CI Width** |
| 7 | 22 | 4475 | 758 | 0.95 | (0.91, 0.97) | 0.06 | 0.61 | (0.52, 0.69) | 0.17 |
| 8 | 22 | 4475 | 758 | 0.92 | (0.87, 0.96) | 0.09 | 0.70 | (0.61, 0.76) | 0.15 |
| 9 | 22 | 4475 | 758 | 0.88 | (0.82, 0.93) | 0.11 | 0.76 | (0.70, 0.82) | 0.12 |
| 10 | 22 | 4475 | 758 | 0.84 | (0.76, 0.90) | 0.14 | 0.82 | (0.76, 0.86) | 0.10 |
| 11 | 22 | 4475 | 758 | 0.77 | (0.69, 0.85) | 0.16 | 0.86 | (0.81, 0.89) | 0.08 |
| 12 | 22 | 4475 | 758 | 0.70 | (0.61, 0.79) | 0.18 | 0.89 | (0.85, 0.92) | 0.07 |
| 13 | 22 | 4475 | 758 | 0.62 | (0.52, 0.72) | 0.20 | 0.91 | (0.88, 0.93) | 0.05 |
| 14 | 22 | 4475 | 758 | 0.53 | (0.44, 0.63) | 0.19 | 0.93 | (0.91, 0.95) | 0.04 |
| 15 | 22 | 4475 | 758 | 0.45 | (0.36, 0.54) | 0.18 | 0.95 | (0.93, 0.96) | 0.03 |

Table A5: Estimated sensitivity and specificity, 95% confidence intervals (CI) and CI widths for each cut-off when Hoyer and Kuss^19^ model is fit to the published (top) and full IPD (bottom) dataset.

| **Hoyer and Kuss^19^ fit to *Published data*** | | | | | | | | | |
| --- | --- | --- | --- | --- | --- | --- | --- | --- | --- |
| **Cut-off** | **N**  **Studies** | **N**  **Participants** | **N**  **MDD Cases** | **Sensitivity** | **(95% CI)** | **Sensitivity CI Width** | **Specificity** | **(95% CI)** | **Specificity CI Width** |
| 7 | 9 | 1829 | 265 | 0.91 | (0.88, 0.95) | 0.07 | 0.61 | (0.55, 0.68) | 0.13 |
| 8 | 11 | 2336 | 337 | 0.88 | (0.84, 0.92) | 0.08 | 0.69 | (0.63, 0.76) | 0.13 |
| 9 | 14 | 3127 | 460 | 0.85 | (0.80, 0.90) | 0.10 | 0.76 | (0.70, 0.82) | 0.12 |
| 10 | 13 | 2631 | 353 | 0.81 | (0.74, 0.87) | 0.13 | 0.81 | (0.76, 0.87) | 0.11 |
| 11 | 14 | 2782 | 395 | 0.76 | (0.69, 0.83) | 0.14 | 0.86 | (0.81, 0.90) | 0.09 |
| 12 | 13 | 2693 | 370 | 0.71 | (0.62, 0.79) | 0.17 | 0.89 | (0.85, 0.93) | 0.08 |
| 13 | 18 | 3398 | 568 | 0.65 | (0.56, 0.75) | 0.19 | 0.91 | (0.88, 0.95) | 0.07 |
| 14 | 10 | 2326 | 265 | 0.60 | (0.49, 0.70) | 0.21 | 0.93 | (0.91, 0.96) | 0.05 |
| 15 | 6 | 1286 | 131 | 0.54 | (0.43, 0.66) | 0.23 | 0.95 | (0.93, 0.97) | 0.04 |
| **Hoyer and Kuss^19^ fit to *full IPD*** | | | | | | | | | |
| **Cut-off** | **N**  **Studies** | **N**  **Participants** | **N**  **MDD Cases** | **Sensitivity** | **(95% CI)** | **Sensitivity CI Width** | **Specificity** | **(95% CI)** | **Specificity CI Width** |
| 7 | 22 | 4475 | 758 | 0.96 | (0.95, 0.98) | 0.03 | 0.56 | (0.49, 0.62) | 0.13 |
| 8 | 22 | 4475 | 758 | 0.94 | (0.92, 0.96) | 0.04 | 0.63 | (0.57, 0.69) | 0.12 |
| 9 | 22 | 4475 | 758 | 0.91 | (0.88, 0.93) | 0.05 | 0.69 | (0.63, 0.75) | 0.12 |
| 10 | 22 | 4475 | 758 | 0.86 | (0.83, 0.90) | 0.07 | 0.74 | (0.69, 0.80) | 0.11 |
| 11 | 22 | 4475 | 758 | 0.81 | (0.77, 0.85) | 0.08 | 0.79 | (0.73, 0.84) | 0.11 |
| 12 | 22 | 4475 | 758 | 0.75 | (0.70, 0.80) | 0.10 | 0.82 | (0.77, 0.87) | 0.10 |
| 13 | 22 | 4475 | 758 | 0.68 | (0.62, 0.74) | 0.12 | 0.85 | (0.81, 0.89) | 0.08 |
| 14 | 22 | 4475 | 758 | 0.61 | (0.54, 0.67) | 0.13 | 0.87 | (0.84, 0.91) | 0.07 |
| 15 | 22 | 4475 | 758 | 0.53 | (0.46, 0.60) | 0.14 | 0.90 | (0.86, 0.93) | 0.07 |


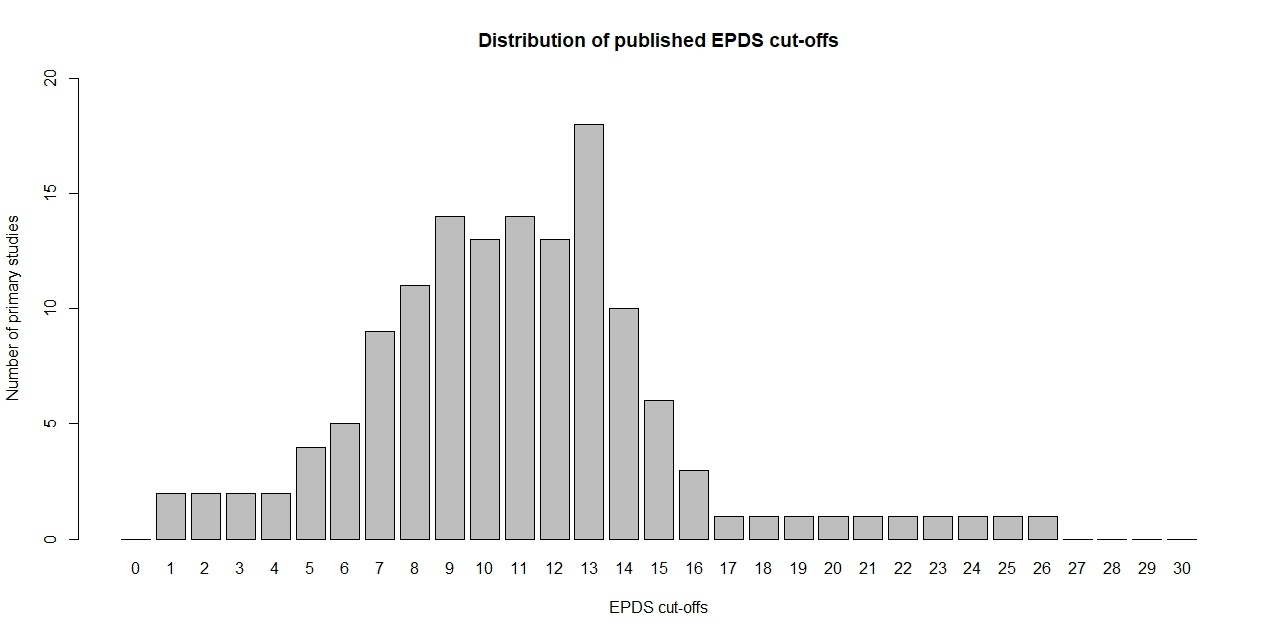


Figure A1: Distribution of published EPDS cut-offs by the number of primary studies included in the meta-analyses using the *published dataset*.


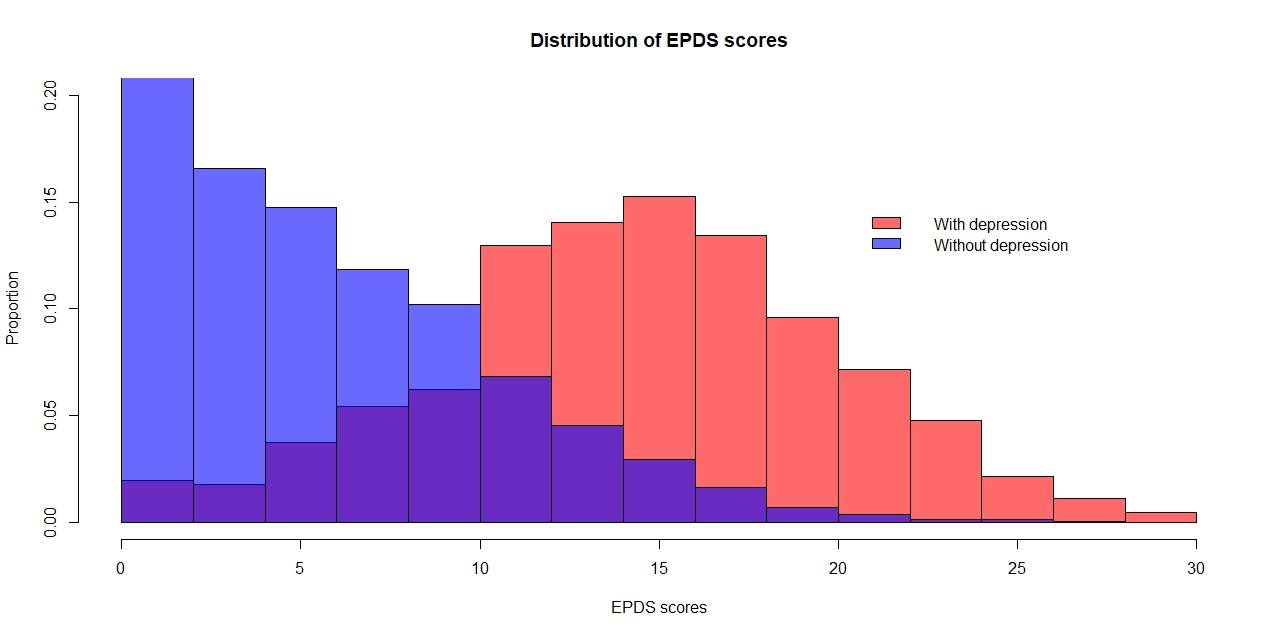


Figure A2: Distribution of EPDS scores among participants with depression (red) and without depression (blue). Purple portions are part of both the blue and red distributions.


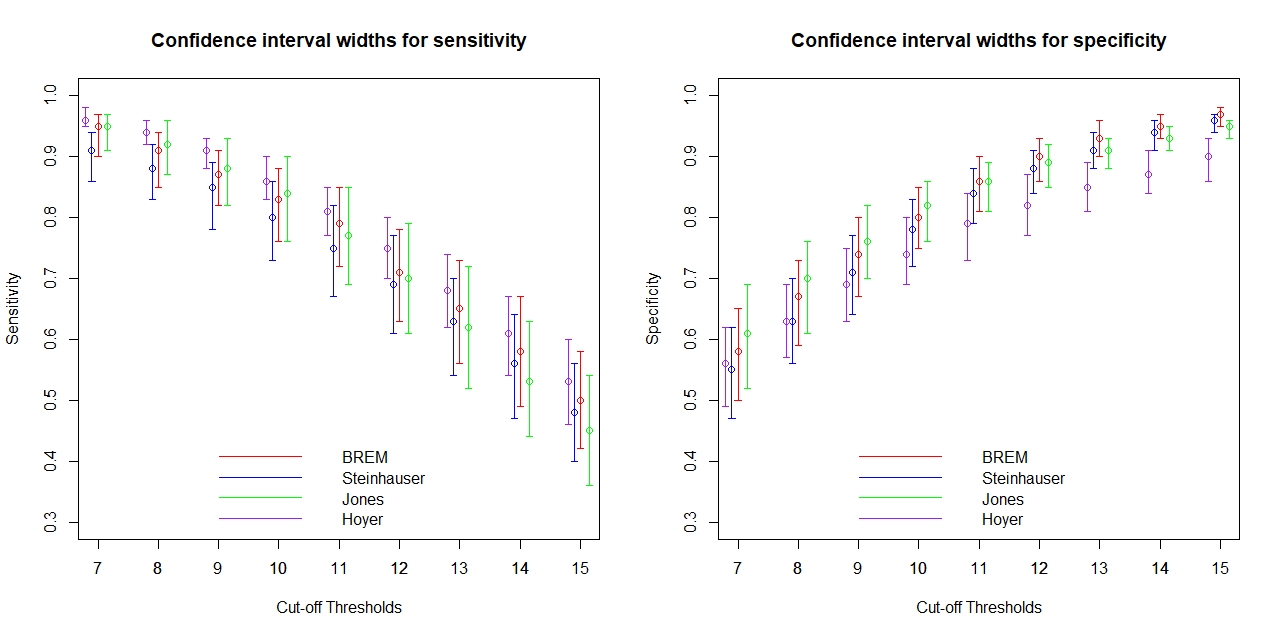


Figure A3: Estimated sensitivity (left) and specificity (right) and 95% Confidence Interval (Credible Interval for Jones et al.^18^) by cut-off for the BREM,**^13^** Steinhauser et al.,^16^ Jones et al.^18^ and Hoyer and Kuss^19^ methods applied to the full IPD dataset.


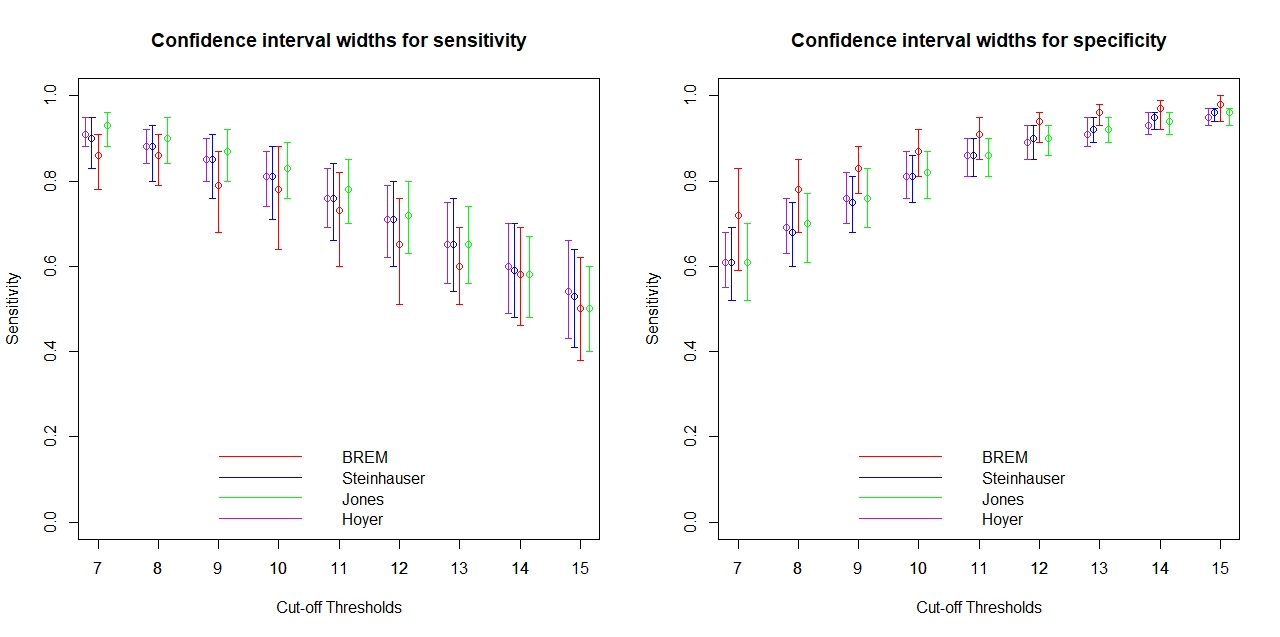


Figure A4: Estimated sensitivity (left) and specificity (right) and 95% Confidence Interval (Credible Interval for Jones et al.^18^) by cut-off for the BREM,^13^ Steinhauser et al.,^16^ Jones et al.^18^ and Hoyer and Kuss^19^ methods applied to the published dataset.
